# Nanoporous Films with Oriented Arrays of Molecular Motors for Photoswitching the Guest Adsorption and Diffusion

**DOI:** 10.1002/anie.202214202

**Published:** 2022-12-20

**Authors:** Yunzhe Jiang, Wojciech Danowski, Ben L. Feringa, Lars Heinke

**Affiliations:** ^1^ Institute of Functional Interfaces (IFG) Karlsruhe Institute of Technology (KIT) 76344 Eggenstein-Leopoldshafen Germany; ^2^ Centre for Systems Chemistry Stratingh Institute for Chemistry University of Groningen 9747 Nijenborgh 4 Groningen, AG The Netherlands; ^3^ University of Strasbourg CNRS ISIS UMR 7006 8 Allée Gaspard Monge 67000 Strasbourg France

**Keywords:** Diffusion, Metal–Organic Framework, Overcrowded Alkene, Photoswitches, Uptake

## Abstract

Molecular motors are fascinating nanomachines. However, constructing smart materials from such functional molecules presents a severe challenge in material science. Here, we present a bottom‐up layer‐by‐layer assembly of oriented overcrowded‐alkene molecular motors forming a crystalline metal–organic framework thin film. While all stator parts of the overcrowded‐alkene motors are oriented perpendicular to the substrate, the rotors point into the pores, which are large enough allowing for the light‐induced molecular rotation. Taking advantage of the thin film's transparency, the motor rotation and its activation energy are determined by UV/Vis spectroscopy. As shown by gravimetric uptake experiments, molecular motors in crystalline porous materials are used, for the first time, to control the adsorption and diffusion properties of guest molecules in the pores, here, by switching with light between the (meta‐)stable states. The work demonstrates the potential of designed materials with molecular motors and indicates a path for the future development of smart materials.

## Graphical abstract

A regular array of oriented molecular motors is presented by incorporating overcrowded alkene molecules as linker in metal–organic framework thin films. By switching between the stable and metastable states, the adsorption and diffusion properties of the guest molecules are controlled.

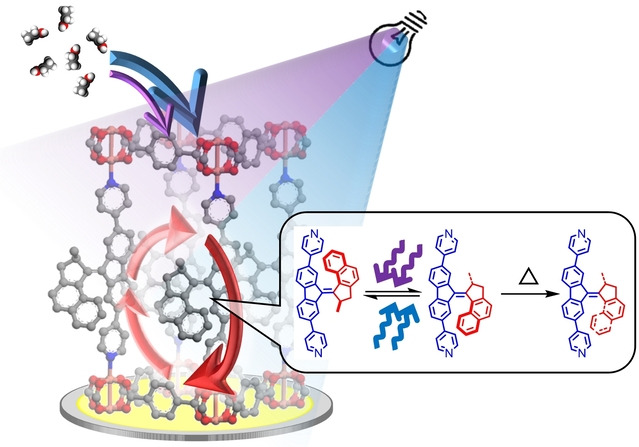

Inspired by molecular machines and molecular motors in natural biological cells, artificial molecular machines draw great scientific attention.^[1]^ This resulted in significant progress during the last decades and various artificial molecular motors were presented.^[2]^ Particularly fascinating artificial molecular motors are overcrowded alkene‐based molecular motors, which are composed of a motor and stator part, connected by an alkene bond.^[3]^ The molecule performs a unidirectional 360° rotation, composed of four steps, Figure 1a. Two steps, i.e. step 1→2 and 3→4, are powered by light, leading to isomerization of the molecule from the stable to the metastable state. The other two steps, i.e. step 2→3 and 4→1, are thermal relaxation, referred to as thermal helix inversion (THI), from the metastable to the consecutive stable state, completing the molecular rotation.[^3e^, ^4^] This unique molecular characteristic enables various potential applications in solvents, gels and surface coatings, showing both mechanical and chemical changes from the nano to the macro scale.^[5]^ In most demonstrations, the molecular motors are incorporated in polymers or in solutions to avoid steric hindrance, prohibiting the molecular rotation.^[6]^ However, in solution or polymers, the molecular rotors are not oriented, hindering a collective directional rotation of the material. The steric hindrance of the molecular rotation can also be avoided by their incorporation in porous materials, like metal–organic frameworks (MOFs).^[7]^ MOFs are solid porous materials made of metal nodes connected by organic linker molecules.^[8]^ MOFs have a regular, crystalline structure with uniform pores. MOFs with incorporated or embedded photo‐switchable molecules such as azobenzene or spiropyran have been explored.^[9]^ By exchanging a large percentage of linker molecules of the MOF by functionalized overcrowded‐alkene‐motor molecules, MOF powders with a high ratio of incorporated molecular motors were presented.[^7^, ^10^] The pores can provide sufficient space for each motor molecule to rotate. Apart from the presentation of regular, crystalline arrays of molecular motors, MOF materials where molecular motors perform a (macroscopic) functionality, like changing the guest uptake capacity, have not yet been presented. Due to the short penetration depth of the incident light, only the outer shell of the MOF particles and powders can be illuminated, and the region of rotating molecular motors is limited. To ensure the entire illumination of the material, MOFs can be made in form of homogeneous, well‐defined thin films using a layer‐by‐layer method. Such MOF films are referred to as surface‐mounted metal–organic frameworks (SURMOFs).^[11]^ The high transparency of the SURMOFs^[12]^ enable their exploration via UV/Vis spectroscopy in transmission mode,[^11a^, ^13^] which was employed to explore the photo‐response of various MOF materials.[^13^, ^14^] By optimized substrate functionalization, the crystalline orientation of the SURMOF film and of the molecular components can be controlled.^[15]^

**Figure 1:**
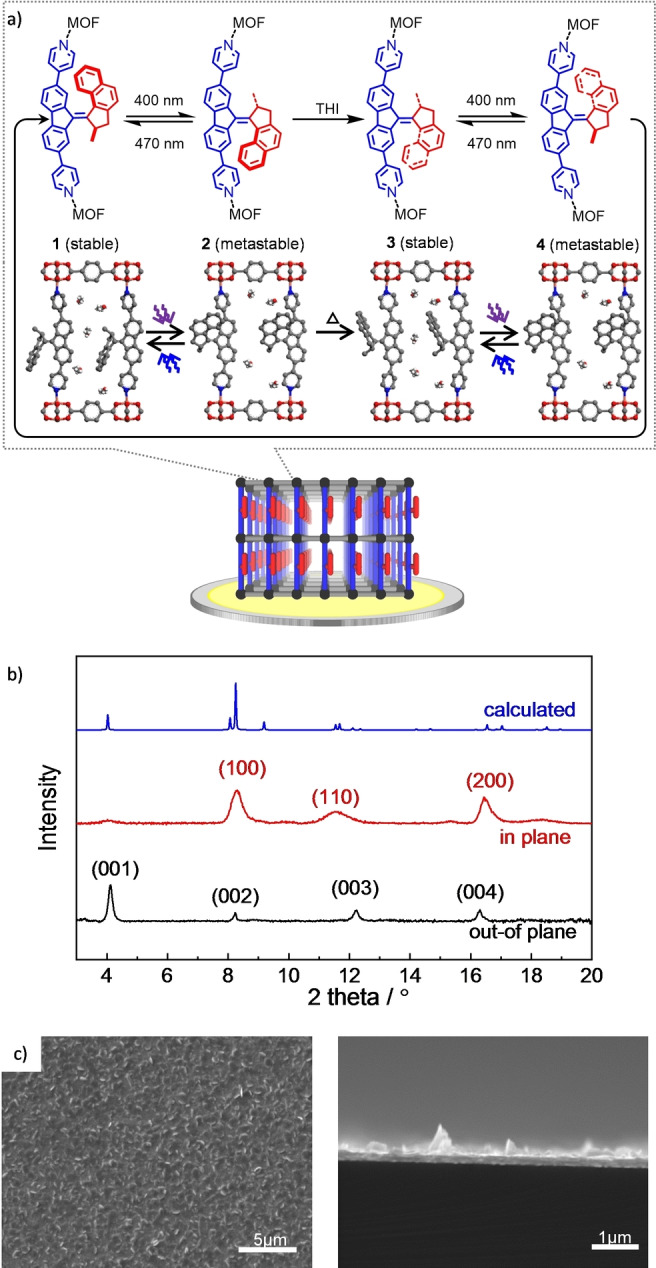
a) Overcrowded‐alkene molecular motor, referred to as motor‐Py, and the structure of the Cu_2_(BDC)_2_(motor‐Py) SURMOF. The motor‐Py molecule is composed of a molecular stator (blue) and rotor (red) part. Upon light irradiation of 400 nm wavelength, the motor‐Py molecule undergoes isomerization from the stable state (1 and 3) to the metastable state (2 and 4). Upon thermal relaxation by thermal helix inversion (THI), the molecule reaches the consecutive stable state (3 and 1) and the rotor part rotates by 180°. Upon another cycle, the rotor performs one full rotation (1→2→3→4→1). In addition to the forward rotation, the molecule in the metastable state can be switched back to the initial diastereomer by irradiation with light of 470 nm. The change of the adsorption capacity of ethanol molecules in the SURMOF in different states is sketched. b) Out of‐plane XRD (black), in‐plane XRD (red) and the calculated XRD (blue) of Cu_2_(BDC)_2_(motor‐Py) SURMOF. The experimentally observed reflexes are labelled. The X‐ray wavelength is 0.154 nm. c) SEM images of the SURMOF sample on gold substrate. (Left: top‐view, right: cross‐section of broken sample).

Here, we present an oriented array of molecular motors. This material is based on oriented, highly crystalline SURMOF films with a pillared‐layer structure. The pillar moieties of the SURMOF structure, which are oriented perpendicular to the substrate, are the stators of the molecular overcrowded alkene motor. The rotor parts of the motor point into the pores and performs light‐fueled rotations (Figure 1 and S1). Enabled by the structural quality of the SURMOF films, the light‐induced molecular rotation is explored by UV/Vis spectroscopy in transmission mode. Temperature‐dependent experiments allow the quantification of the rate of the thermal helix inversion, as a limiting step for the molecular rotation. By gravimetric guest uptake experiments using a quartz crystal microbalance (QCM), the adsorption capacity and the diffusion coefficient of the guest molecules, here ethanol, were quantified. For the first time, the impact of the different states of the molecular rotor (see Figure 1a) on the uptake amount and mass transfer was quantified.

The overcrowded alkene motor molecule, termed motor‐Py, is composed of a stator and a rotor part, Figure 1a. The compound was synthesized as described in ref. [7]. The stator part is terminated by two pyridyl groups, making it perfectly suited for the incorporation in pillared‐layer MOF structures. The synthesis of the SURMOF was performed in a layer‐by‐layer fashion by subsequently soaking the modified substrate into the ethanolic solutions of the MOF components, which are copper acetate (copper paddle wheels) solution and terephthalic acid (BDC) and motor‐Py solution, see also Supporting Information.

The X‐ray diffractograms (XRDs, Figure 1b) show that the SURMOF possesses a highly crystalline structure, which corresponds to the targeted pillared‐layer Cu_2_(BDC)_2_(motor‐Py) structure, see Figures 1a and S1.^[16]^ The out‐of‐plane XRD data show only a series of [00n] peaks, which indicates that the SURMOF is grown in a highly oriented fashion. The in‐plane XRD, which measures the crystallinity in the direction perpendicular to the surface normal, shows reflexes of the [100] and [110] directions, which are perpendicular to [001]. Both data, from in‐plane and from out‐of‐plane XRD, clearly indicate that the SURMOF is grown in a highly oriented fashion, where the BDC linkers form layers parallel to the substrate surface and the motor‐Py molecules are perpendicular to the surface. This means the stators of the molecular motor are standing perpendicular on the substrate, as sketched in Figure 1a.

The scanning electron microscopy (SEM) pictures, Figures 1c and S2, show that the SURMOF is a homogeneous dense film with a thickness of approximately 200 nm. Small crystallites and flakes on top of the film can also be observed.

The molecular isomerization resulting in the molecular rotation of the motor‐Py component in the SURMOF is characterized by UV/Vis spectroscopy, Figure 2. In the UV/Vis spectra, absorption intensity changes at around 420 nm can be observed upon violet light irradiation. Compared with the spectra of motor‐Py in solution (see Figure S5), the absorption bands are red‐shifted in the SURMOF, this means the bands are shifted from 390 nm to 415 nm and from 448 nm to 488 nm, respectively. The red shift of the peak position is a clear evidence that the molecular energy levels of the motor molecule shift when incorporated in the SURMOF, most likely due to the coordination of the stator to the copper paddle wheels. To analyze the spectra more precisely, the differential spectra are shown, Figure 2b. The spectral changes upon irradiation with light of 400 nm can be unequivocally correlated to the light‐induced isomerization from the stable state to the metastable state. The spectra are in very good agreement with detailed spectroscopic studies in solution.[^7^, ^17^] In line with these studies, the spectra changes upon irradiation with 470 nm are correlated to the isomerization from the metastable state (e.g. state 2) back to the stable state (e.g. state 1). Please note, by using light for the isomerization to a stable ground state, the rotor rotates back to the initial position and no (forward) rotation is performed. The (forward) rotation is performed by THI in the dark, see Figure 1a. To explore the reversibility of the motor‐Py isomerization in the SURMOF, a series of irradiation with various illumination times was carried out, see inset in Figure 2b. The data show that the ideal irradiation time for light of 400 nm (resulting in the largest reversible spectra change) is approximately 1 min, while the time for the 470 nm irradiation is approximately 6 min. Longer irradiation does not enhance the isomerization yield or rotation performance, but leads to some photobleaching.

**Figure 2:**
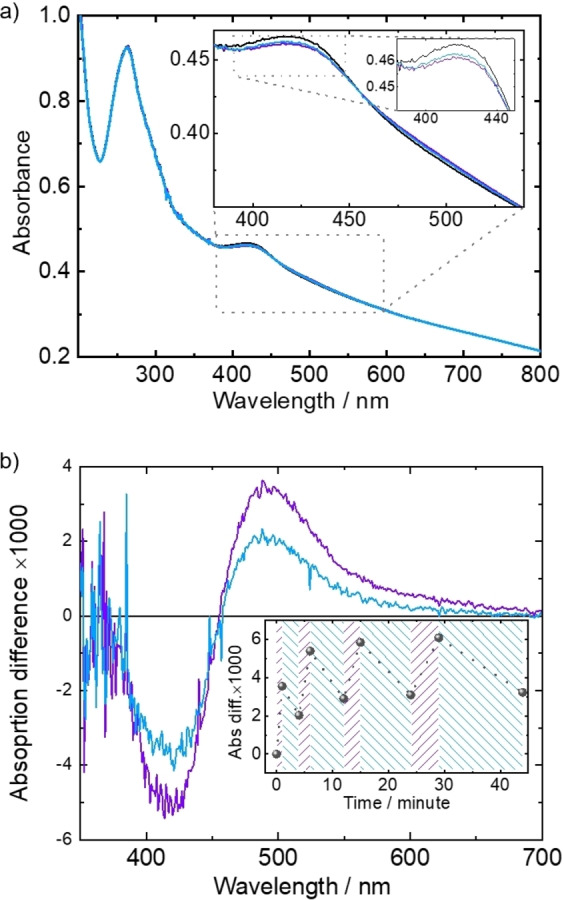
a) UV/Vis spectra of Cu_2_(BDC)_2_(motor‐Py) SURMOF: before (black), after 400 nm light irradiation (violet) and after 470 nm light irradiation (blue). The in‐sets show zoom‐ins of the spectra. b) The absorbance difference by subtracting the initial spectrum from the irradiated spectra (both 400 and 470 nm). The in‐set shows the absorbance difference at 488 nm for different irradiation time. The 400 nm irradiation lasts 1, 2, 3 and 5 min while the 470 nm irradiation lasts 3, 6, 9 and 15 min, respectively. The full spectra are shown in Figure S3.

The thermal helix inversion (THI) behavior of the motor inside the SURMOF was explored by measuring the transient UV/Vis spectra. In the transient spectra (Figure S4, left) and in the differential spectra (Figure 3a and S4, middle), it can be clearly seen that the light‐induced changes of the absorption bands disappear with time, in agreement with the thermal isomerization of the metastable diastereomer. Please note, the data show that a quantitative switching back to the pristine state by light or THI was not realized.

**Figure 3:**
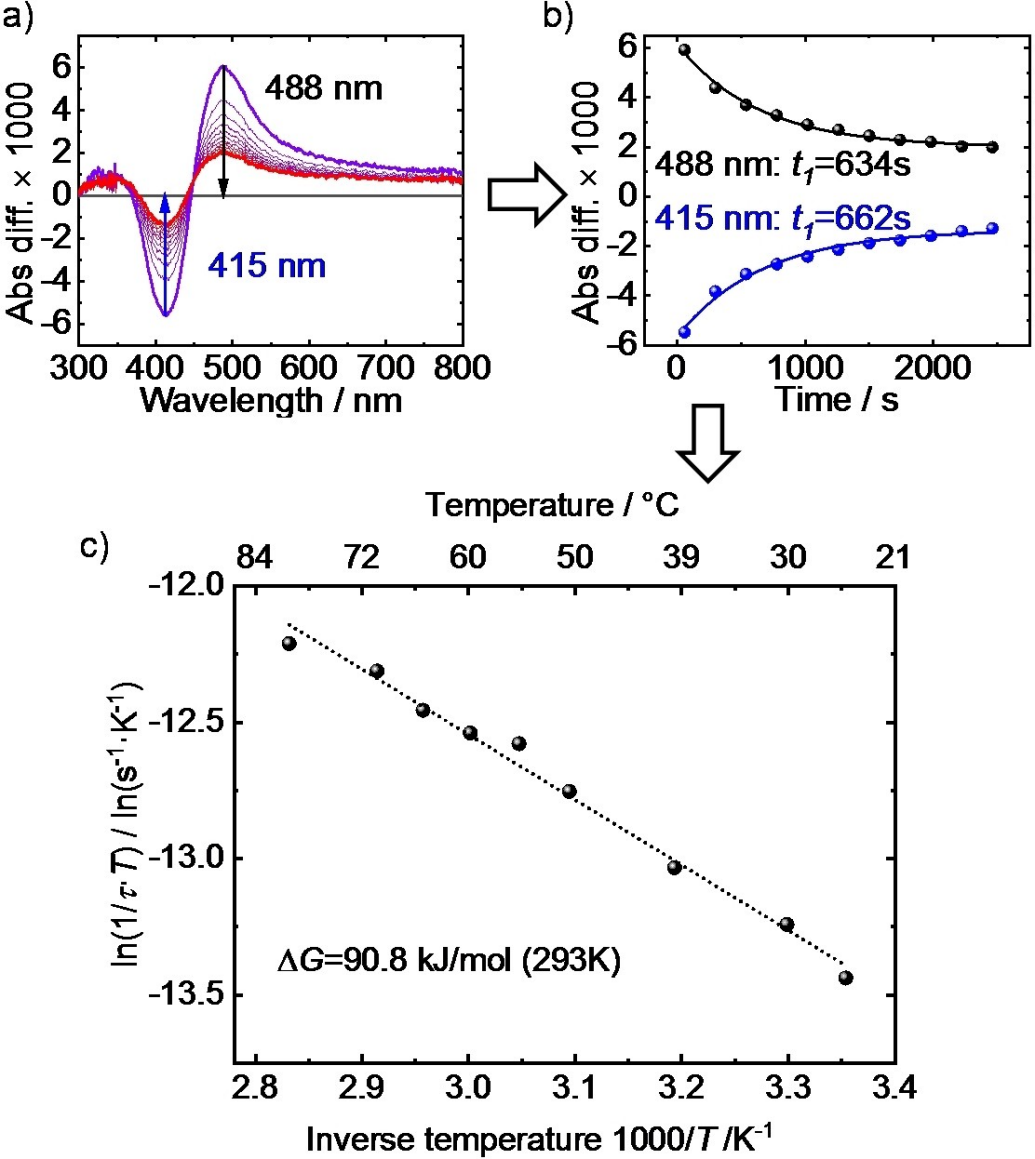
a) Differential spectra of the Cu_2_(BDC)_2_(motor‐Py) SURMOF. The black spectrum is from the thermally relaxed sample and violet after 400 nm‐irradiation, followed by the isomerization from the metastable state to the consecutive stable state (THI). The thin lines are the spectra upon the 400 nm‐irradiation with a time difference of 240 s. The experiments are performed at 70 °C. b) The absorbance difference of the UV/Vis bands at 488 nm (black) and 415 nm (blue) as function of time. The fits with the mono‐exponential decay functions and the determined time constants are shown. c) Eyring plot of the time constants determined from the mono‐exponential decay functions in panel b) and Figure S4. The time constants are the average from the decay of the bands at 488 nm and 415 nm (see panel b). The line is the fit with the Eyring equation, ln(1/(*τT*))=ln(*k*_B_/*h*)−Δ*H* ^≠^/*RT*+Δ*S* ^≠^/*R*, where *τ* is the time constant, *T* is the temperature, *k*_B_ is the Boltzmann's constant, *h* is the Planck's constant, Δ*H* ^≠^ is the enthalpy of activation, *R* is the universal gas constant and Δ*S* ^≠^ the is entropy of activation. The Gibbs free energy Δ*G* ^≠^(*T*) is calculated by Δ*G* ^≠^(*T*)=Δ*H* ^≠^−*T*Δ*S* ^≠^.

The absorbance differences at the characteristic bands of the overcrowded alkene (Figure 3b and S4) show mono‐exponential decay behaviors with characteristic time constants *τ* (=1/*k*), indicating that the THI‐relaxation is a first‐order reaction. At 25 °C, the (mono‐exponential) time constant is approximately 2300 s, corresponding to a half‐life time of ≈27 min. An Eyring plot of the temperature‐dependent time constants (Figure 3c) show a linear behavior. The determined Gibbs free energy for the THI‐relaxation of motor‐Py incorporated in the MOF is 90.8±1.5 kJ mol^−1^ at 20 °C, which is in agreement with a previous report on the motor in MOFs.^[7]^

For comparison, the motor‐Py molecule dissolved in ethanol solution was measured with the same method (Figure S5). A half‐life time of ≈12 min and a Gibbs free energy of 90.3±1.5 kJ mol^−1^ were determined, which are similar to previous reports.[^7^, ^18^] Noteworthy, the Gibbs free energy of the THI process in the SURMOF (in nitrogen) is similar to that of the free molecule in ethanolic solution, while the life times are different. This is also consistent with ref. [7], where very similar values for the Gibbs free energy of the THI process in the MOF, in various solvents as well as calculated by density‐functional theory were found.

Based on the time constant of the THI which is the rate limiting process for the rotation, we can determine the rotation frequency of the molecular motor in the SURMOF. Each full rotation comprises of two THI processes. While the full rotation of the rotor part takes about 1 h (2 times the characteristic half‐life time) at room temperature, the full rotation takes approximately 15 min at 70 °C.

In the next step, we take advantage of the photo‐induced isomerization of the motor‐Py molecule in the SURMOF. To this end, the SURMOF was prepared on gold‐coated QCM sensors and the transient uptake of a probe molecule, here ethanol, was gravimetrically determined.^[19]^ Initially, the SURMOF was in an atmosphere of pure nitrogen and the pores were empty. At *t*=0, the pure nitrogen flow was switched to the nitrogen flow enriched with ethanol with an almost saturated vapor pressure.^[20]^ Figure 4a shows the transient uptakes by the sample in the pristine state and upon light irradiation, this means in the stable state and metastable state. In all uptake experiments, the final equilibrium loading in the pores was reached after a few seconds, regardless of the state, i.e. stable or metastable. Upon 400 nm irradiation, this means switching the SURMOF from the stable state to the metastable state, the equilibrium loading decreases from 1.37 μg cm^−2^ to 1.12 μg cm^−2^, i.e. by about 19 %. Upon 470 nm irradiation, the uptake amount increases again. 3 cycles of 400 nm and 470 nm irradiation were performed, showing similar results, Figure 4b and S6. This indicates a good stability of the sample and reproducible uptake changes. The different adsorption capacities (in the stable versus meta‐stable state) can be explained by the different surface areas of the MOF structures and the different accessible pore volumes. While the calculated specific surface of the MOF in the stable state is 3520 m^2^ g^−1^, the calculated specific surface in the metastable state is only 3343 m^2^ g^−1^, i.e. 5 % smaller, see Supporting Information, table S1. The accessible pore volume per MOF unit cell also changes from 0.486 nm^3^ to from 0.429 nm^3^, i.e. by 12 %. This is consistent with the observed changes of the ethanol uptake.

**Figure 4:**
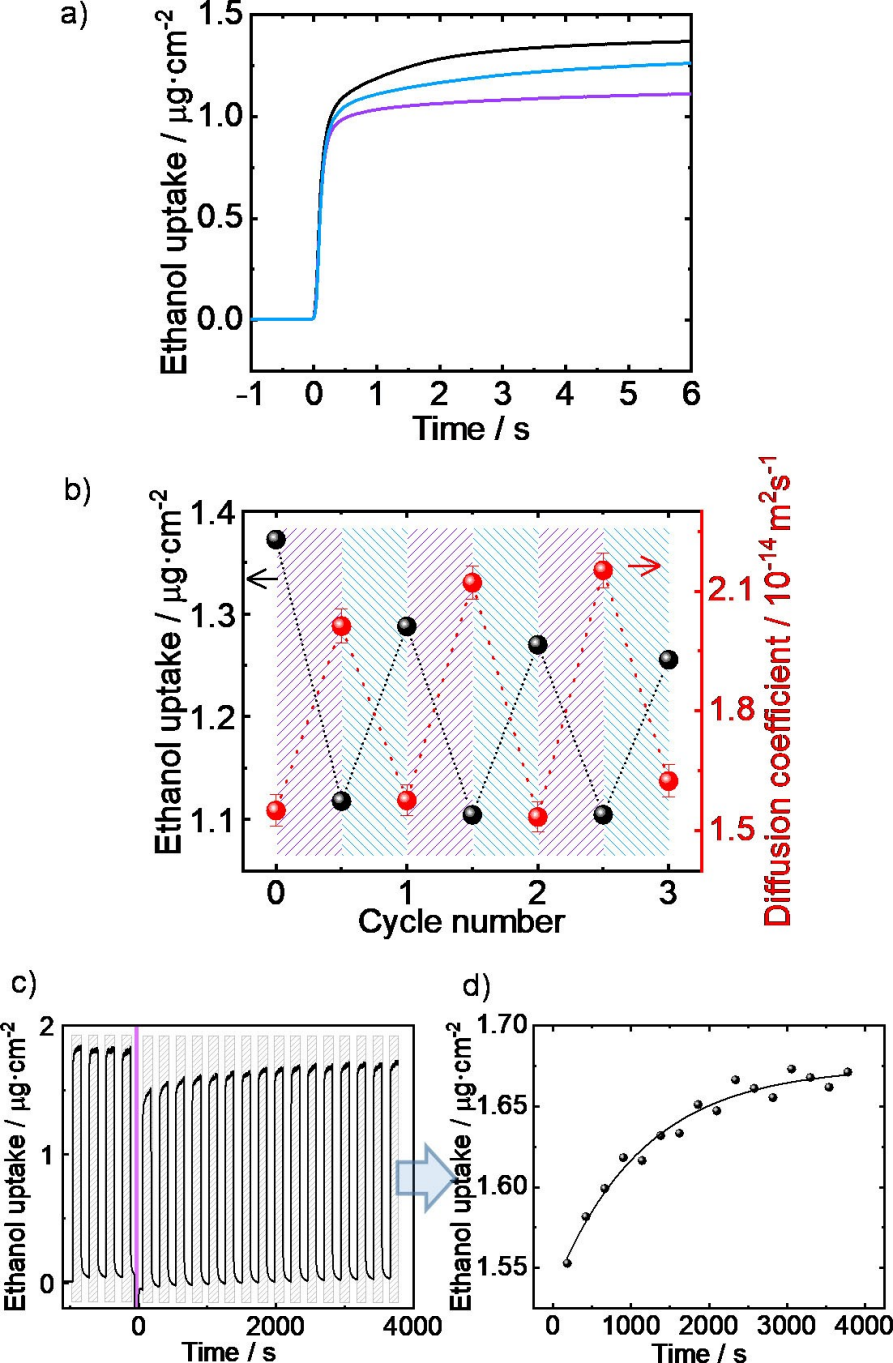
a) Ethanol uptake change of Cu_2_(BDC)_2_(motor‐Py) SURMOF during photoswitching: before (black), after 400 nm light irradiation (violet) and after 470 nm light irradiation (blue). b) The change of the uptake amount (black, scale on the left) and of the diffusion coefficient (red, scale on the right) for 3 cycles of irradiation of 400 (violet) and 470 nm (blue). The error bars of the diffusion coefficients are from the fit accuracy. For more details, see Figures S6 and S7. c) Ethanol uptake change of Cu_2_(BDC)_2_(motor‐Py) SURMOF during THI relaxation: Before and after the irradiation with violet light for 1 min (violet bar), ethanol‐enriched N_2_ and pure N_2_ was alternately flowing through the QCM cell every two min. The grey shadow shows the exploration to ethanol vapor. d) The uptake amount of ethanol in each cycle in Figure 4c. The data is described by a mono‐exponential decay function with a time constant of 1147 s.

In the plots of the transient uptake (Figures 4a and S6), it is visible that the final (equilibrium) value of the uptake is reached somewhat faster for the sample in the metastable state (upon 400 nm irradiation) than for the sample in the stable state (upon 470 nm irradiation). A detailed analysis of the mass transfer kinetics was performed by fitting the experimental data with the solution of Fick's 2^nd^ law for a thin homogeneous film.[^19^, ^21^] The analysis (Figure S7) results in the diffusion coefficients, which are shown in Figure 4b. It shows that the diffusion coefficient of ethanol in the SURMOF in the stable state is approximately 1.5×10^−14^ m^2^ s^−1^, where the diffusion coefficient in the metastable sample is 2.1×10^−14^ m^2^ s^−1^, this means 40 % larger. This switching of the diffusion coefficient is reversible and was repeated for 3 cycles.

In addition, we performed uptake experiments during the THI relaxation, this means during the rotation process, Figure 4c. There, a sample was irradiated with 400 nm and then relaxed in the dark (undergoing THI) at a temperature of 50 °C. During the relaxation process, we performed transient uptake and release experiments with ethanol as probe molecules where each uptake and release step was 2 min, see Figure 4c. After 400 nm irradiation, the uptake amount decreased as in the previous measurements, followed by a gradual increase, indicating that the motor molecule is switching to the stable state. The time constant for retaining the uptake amount was determined to 1147 s (Figure 4d), which is in perfect agreement with the time constant (*τ*) determined by UV/Vis spectroscopy at 50 °C (1070 s, Figure S4d).

In conclusion, a regular array of molecular motors is presented. The array is based on crystalline, nanoporous films of surface‐mounted MOFs with pyridyl‐functionalized overcrowded alkene motors as MOF pillars, standing perpendicular on the substrate surface. The high transparency of the homogeneous MOF film enables the exploration of the isomerization behavior by UV/Vis spectroscopy in transmission mode. There, the time constants of the molecular rotation and the Gibbs free activation energy for the rotation were determined. For the first time, a molecular motor in a nanoporous solid material is used to demonstrate a function. We showed that the uptake capacity and the mass transfer rate, i.e. the diffusion, can be controlled by light. Both properties are modified by switching between the stable and the metastable states of the motor moiety in the SURMOF.

We foresee that such regular and oriented arrays of molecular motors will allow to realize further smart materials with functions like controlled membrane permeation of molecules or ions. We also believe that such SURMOF films can be used as well‐defined model system for advanced spectroscopy, giving insights in the quantum yield^[23]^ and energetic states of the motor,^[24]^ also with different pore fillings, i.e. with different molecular environment for the motor. Moreover, this study of regular arrays of molecular motors may contribute to realize such visionary materials with molecular motors acting as light‐powered “nano‐ventilators”,^[22]^ which direct the molecular motion through the pores.

## Conflict of interest

The authors declare no conflict of interest.

## Supporting information

As a service to our authors and readers, this journal provides supporting information supplied by the authors. Such materials are peer reviewed and may be re‐organized for online delivery, but are not copy‐edited or typeset. Technical support issues arising from supporting information (other than missing files) should be addressed to the authors.

Supporting Information

## Data Availability

The data that support the findings of this study are available from the corresponding author upon reasonable request.
