# Supplementary material for: Nanoporous Films with Oriented Arrays of Molecular Motors for Photoswitching the Guest Adsorption and Diffusion
Source: Angew Chem Int Ed Engl. 2022 Dec 20;62(3):e202214202. doi: 10.1002/anie.202214202 (PMC10107543; doi:10.1002/anie.202214202)
Supplement: Supplementary file 1 — Supporting Information [file ANIE-62-0-s001.pdf]

Angewandte Chemie
Eine Zeitschrift der Gesellschaft Deutscher Chemiker

Supporting Information

Nanoporous Films with Oriented Arrays of Molecular Motors for Photoswitching the Guest Adsorption and Diffusion

*Y. Jiang, W. Danowski, B. L. Feringa, L. Heinke**

Supporting Information

Experimental section

Synthesis of Molecular Motor

The pyridine substituted molecular motor was synthesized according to the previously described procedure.[1]

SURMOF Synthesis

The chemicals were used without further purification: Terephthalic acid (BDC, 98%, Alfa Aesar), copper (II) acetate (99.9% metal, Alfa Aesar), 11-mercapto-1-undecanol (MUD, 99%, Sigma Aldrich) and ethanol (99.5%, VWR Chemicals).

Quartz glass was purchased from GVB GmbH and was washed twice with pure ethanol before using. Gold coated silicon were purchased from Georg Albert PVD coatings. QCM sensors were bought from LOT

$Cu_2(BDC)_2$(motor-Py) SURMOFs were prepared by alternatively exposing the substrates in the ethanolic solutions of the MOF components: 1 mM copper(II) acetate and a mixture of 0.05 mM BDC and 0.05mM motor-Py solution. After each immersion step, the substrate was washed with pure ethanol twice. The substrates are quartz glass functionalized by UV-ozone treatment (for UV-vis measurement), gold-coated silicon wafers functionalized with an 11-mercapto-1-undecanol self-assembled monolayer (for XRD, SEM and Raman), and QCM sensors functionalized with an 11-mercapto-1-undecanol self-assembled monolayer (for ethanol uptake measurement).

X-ray diffraction

Out-of-plane X-ray diffraction (XRD) measurements were carried out with a Bruker D8-Advance diffractometer and a position-sensitive detector in θ-θ geometry. In-plane XRD was measured with a Bruker D8 Discover with a quarter Eulerian cradle, tilt-stage and 2.3° divergence-slits in θ-2θ geometry with a scan step of 0.02°. Both measurements were carried out with Cu-anodes (λ = 0.154 nm) at 40 kV and 40 mA.

Light Irradiation

LEDs with 400 nm (violet) and 470 nm (blue) were used for irradiating the samples. The distance between the sample and LED light was about 1 cm and the power density was roughly 10 $mW{\cdot}cm^{-2}$.

UV-vis spectroscopy

The UV-vis transmission spectra were measured with a Cary5000 spectrometer equipped with an UMA unit from Agilent. For time constant and activation energy calculation, the UV-vis spectra were measured where the sample was placed in a heating cell. The samples (both the SURMOF sample grown on quartz glass and motor-Py solution) were put into a 1 cm × 1 cm cuvette. For the SURMOF measurements, the cuvette was filled with pure nitrogen.

Scanning electron microscopy

Scanning electron microscopy (SEM) images were recorded with a TESCAN VEGA3 tungsten heated filament scanning electron microscope equipped with a Bruker EDX unit. To avoid charging effects, the samples were coated with a thin (~5 nm) platinum film using a LEICA EM ACE600 device.

Ethanol vapor uptake experiments

Ethanol uptake amount was recorded by a quartz crystal microbalance (QCM) system from QSense. N_2 was flowing through an ethanol wash bottle to make ethanol-enriched gas stream. The QCM sensor was put in a QCM cell with a quartz window for light irradiation. Before the measurement, the cell with the sample was purged with pure N_2 at the target temperature for at least 2 hours until a stable baseline was reached, indicating that potential guest molecules desorbed. Before the uptake measurement under irradiation, the ethanol uptake (and release) measurement was performed several times to ensure the stability of the uptake amount. For the THI uptake measurement (Figure 4c), the valve is switched between pure N_2/ethanol-enriched N_2 every 2 minutes. The gas flow rate was always 100 sccm, controlled by a mass flow controller.

Calculation of surface area

The solvent-accessible surface area of the MOF structure in both states were calculated by the “Atom Volumes & Surface” tool with geometry optimized structure with “Forcite calculation” tool in the MaterialStudio software (BIOVIA). The optimization was done in “ultrafine” quality with all parameters set as default. The surface area was calculated with “Fine” grid resolution. Parameter “Max solvent radius” and “Initial solvent radius” were set to 1.82 Å (radius of N_2) while other parameters were used as default. The specific surface area (in m^2/g) were calculated by (accessible solvent surface in m^2)/(Z × f.w. × u)., where Z is the number of asymmetric units in the unit cell (here is 1), f.w. is formula weight (here is 953.95) and u is the unified atomic mass unit (1.6605×10^{-24} g).[2]

Table S1. Accessible solvent surface calculated by material studio (BIOVIA).

	Stable state	Metastable state
Occupied Volume:	2020.04 Å^3	2043.49 Å^3
Free Volume:	486.71 Å^3	429.51 Å^3
Surface Area:	547.01 Å^2	519.51 Å^2
Specific surface area ($m^2\,g^{-1}$)	3520	3344

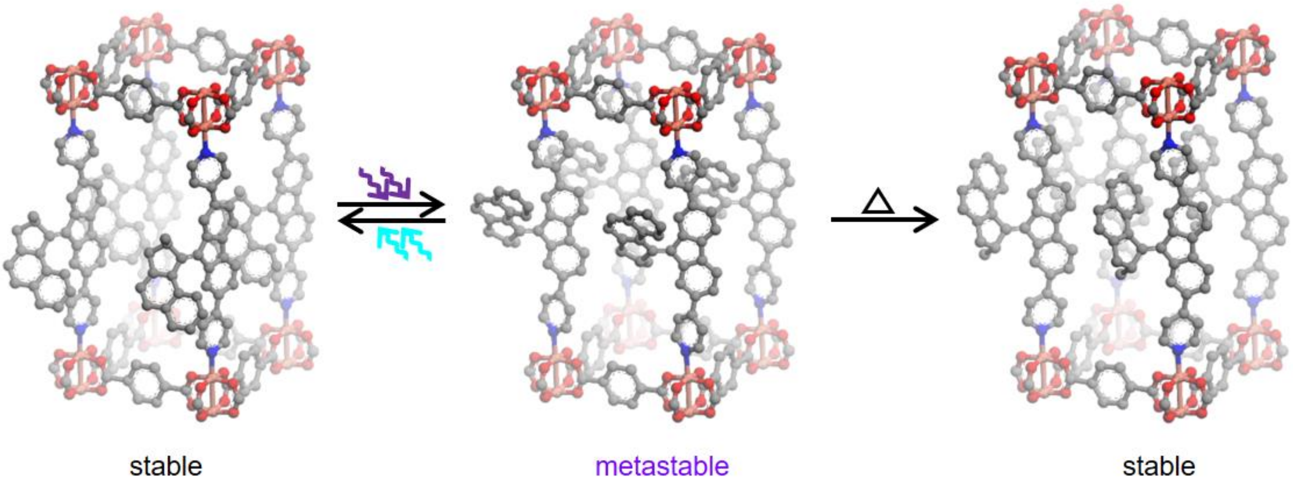

Figure S1. 3d structure of the SURMOF and the rotation of the motor-Py inside the SURMOF during the irradiation and the THI process.[3] Please note, the molecules can rotate around the bonding axes.

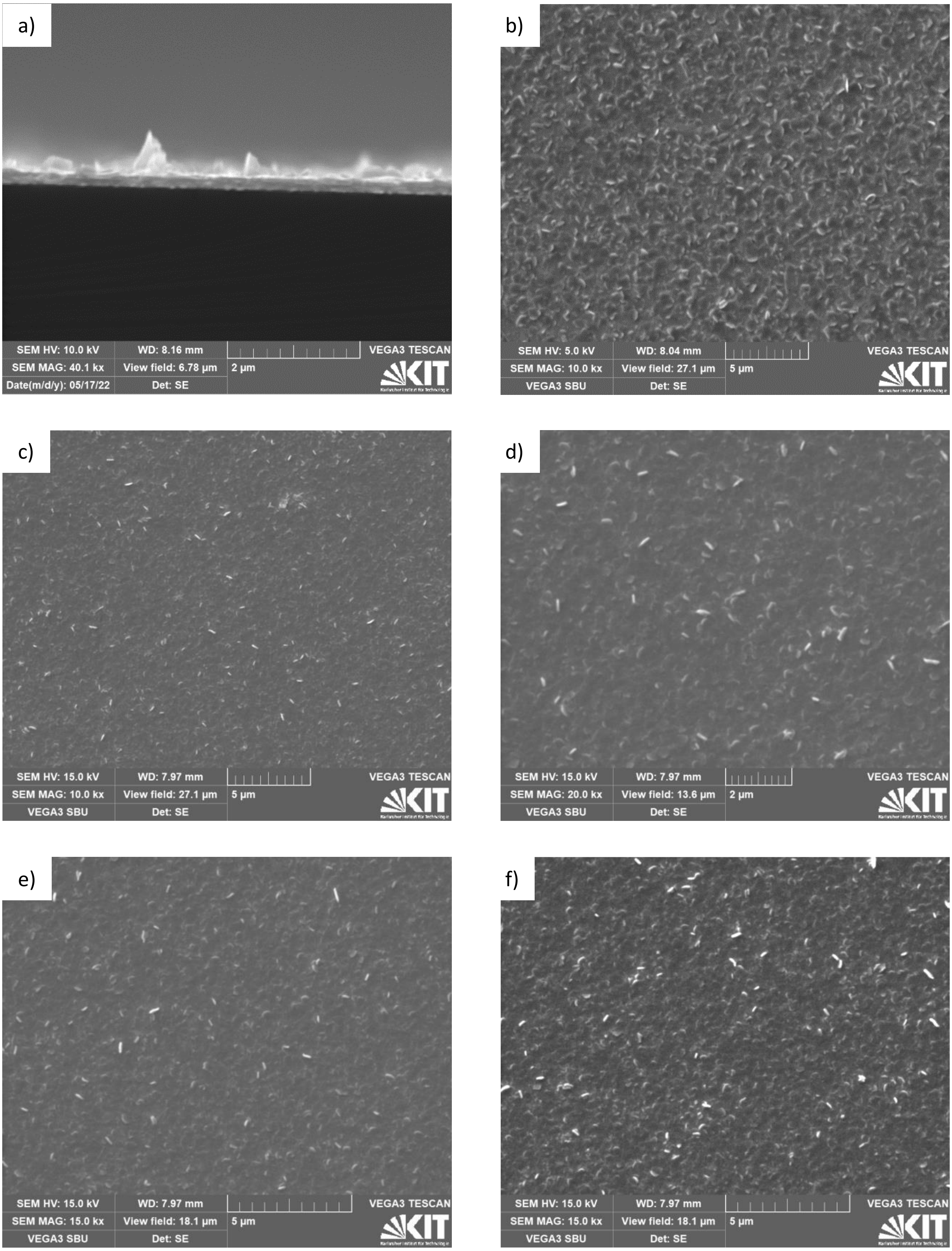

Figure S2. SEM images of the SURMOF sample on the gold substrate. a) Cross-section of the broken sample. b-f) Top view of different regions of the sample. The scan parameters are shown in the pictures.

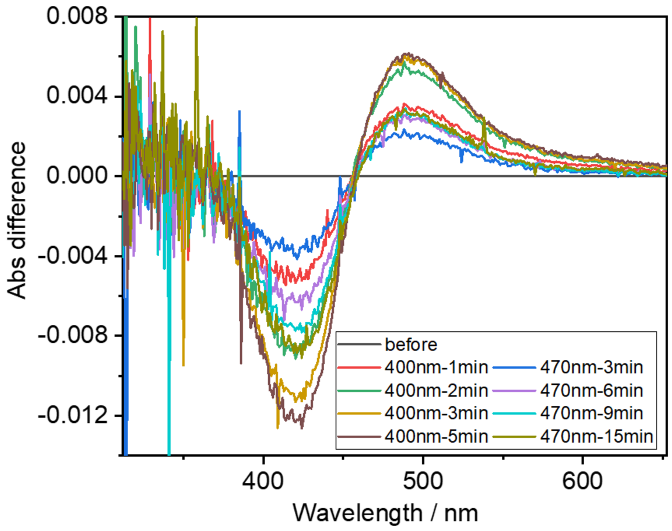

Figure S3. The absorption difference of UV-vis cycle measurement of the $Cu_2(BDC)_2$(motor-Py) SURMOF. The absorption band at 488 nm is used for Figure 2c (inset).

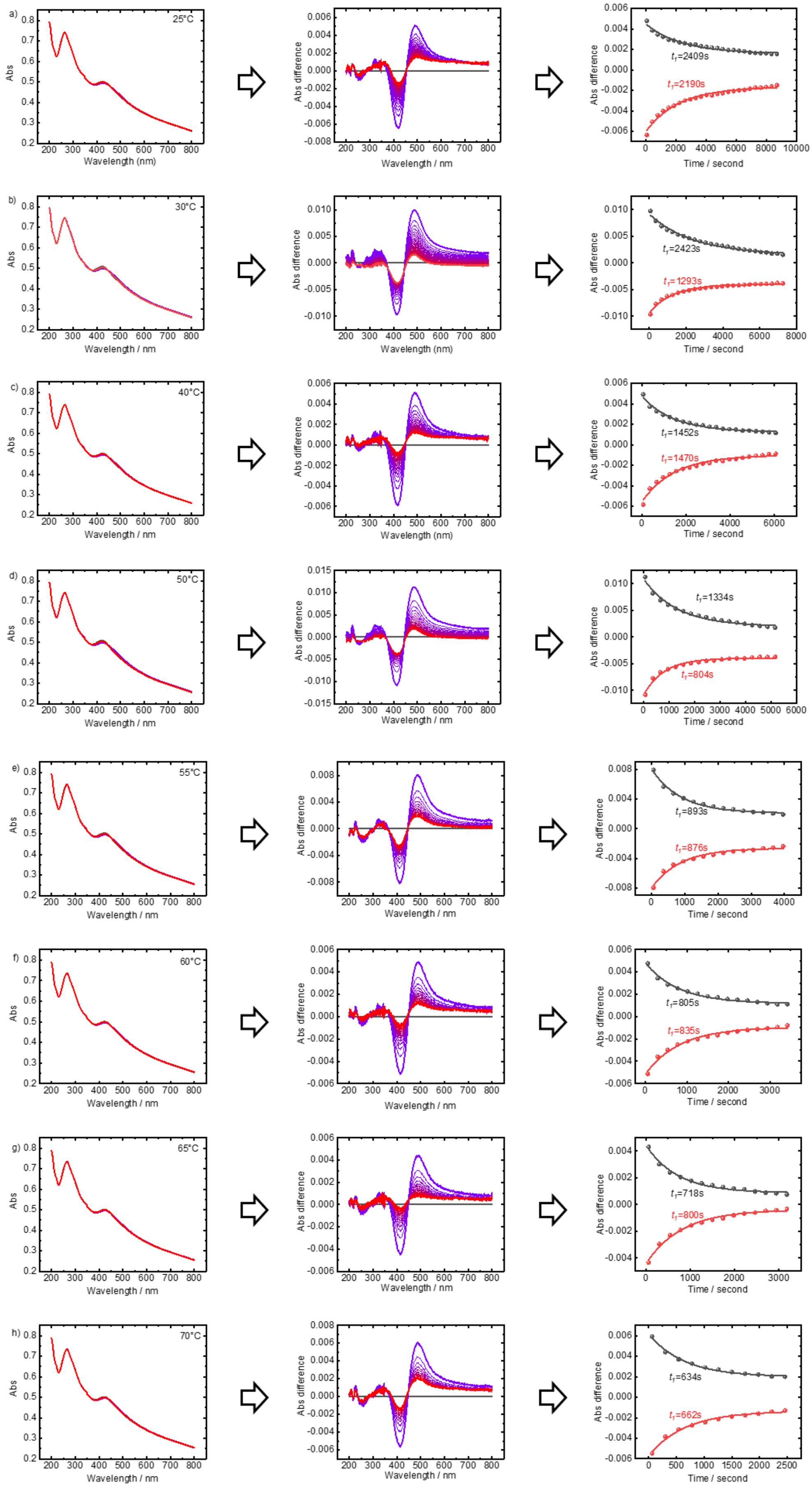

Figure S4. The UV-vis spectra (left) and their difference (center) of $Cu_2(BDC)_2$(motor-Py) SURMOF for THI relaxation at different temperatures. The two peaks (maximum and minimum, see center plots) are chosen to calculate the time constants, and then the average number was used to calculate the Gibbs free energy in fig. 3c. At 25°C, a (mono-exponential) time constant of about 2300 s was determined (average of both values), corresponding to a half-life time of 26.6 min. At 70°C, a time constant of about 650 s was determined, corresponding to a half-life time of 7.5 min.

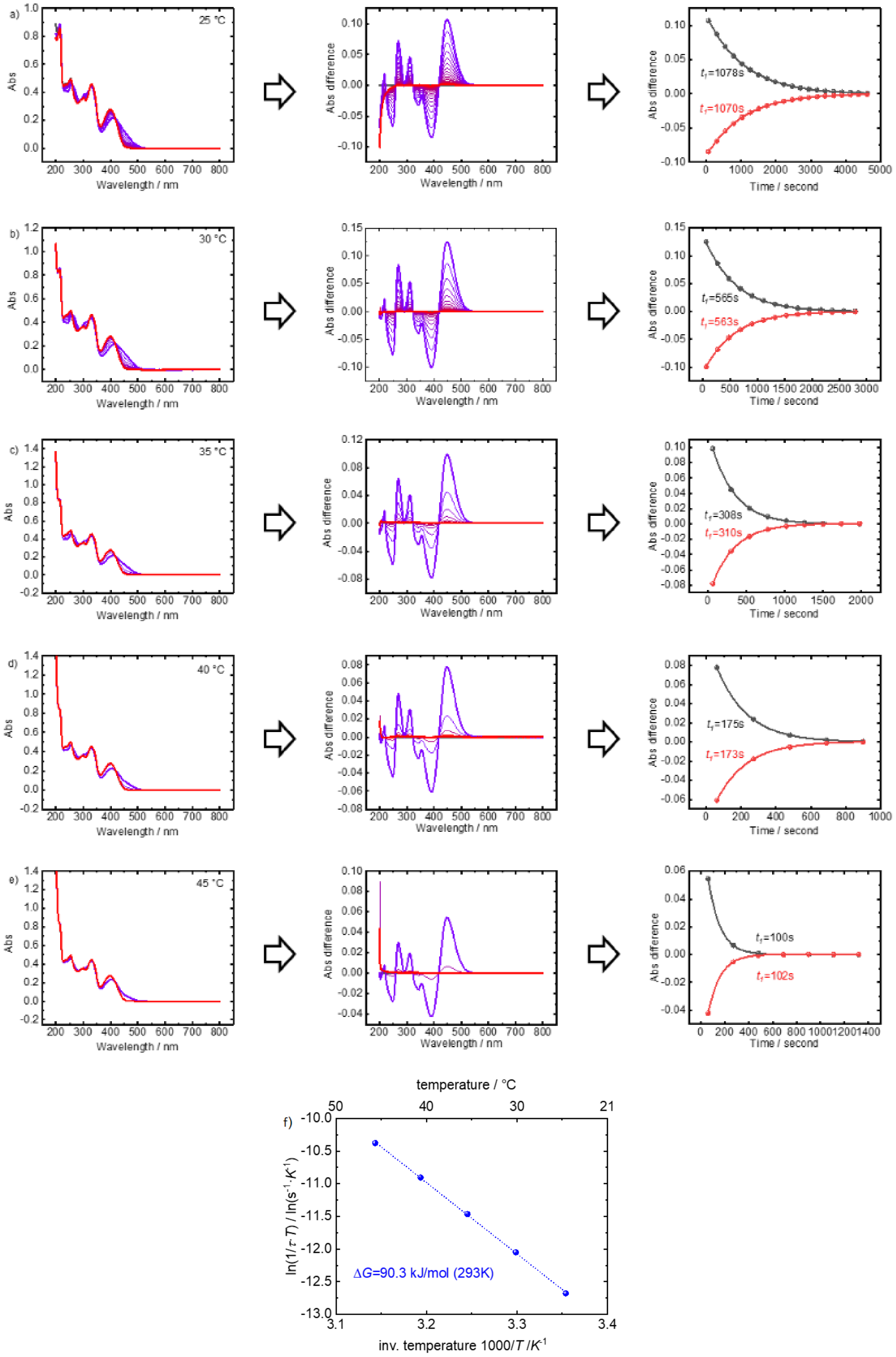

Figure S5. The UV-vis spectra (left) and their difference (center) of the motor-Py linker molecule in ethanol for THI relaxation at different temperatures. The two peaks (maximum and minimum, see center plots) are used to calculate the time constants, and then the average number was used to calculate the Gibbs free energy (panel f). At 25°C, a (mono-exponential) time constant of about 1074 s was determined (average of both values), corresponding to a half-life time of 12.4 min.

The Gibbs free energy of the THI process in the SURMOF (see Figures 3c and S4) and of the free molecule in solution (f) are very similar, which was also found ref.[4] Noteworthy, while the Gibbs free energies are very similar, the activation enthalpies and entropies as well as the time constants vary substantially (see table S2 in ref.[4]), which was also found in our study.

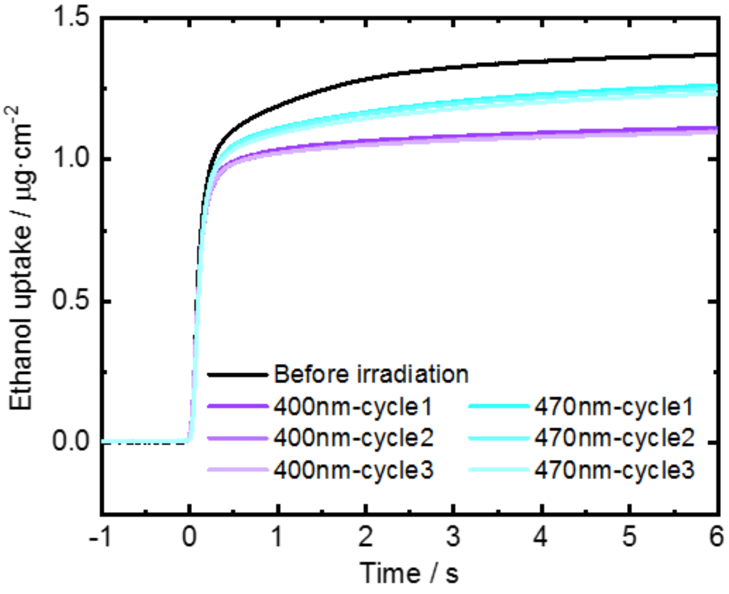

Figure S6. 3 cycles of ethanol uptake, measured by QCM. The first cycle is shown in Figure 4a and the adsorption amount at a time of 6 s was used for Figure 4b.

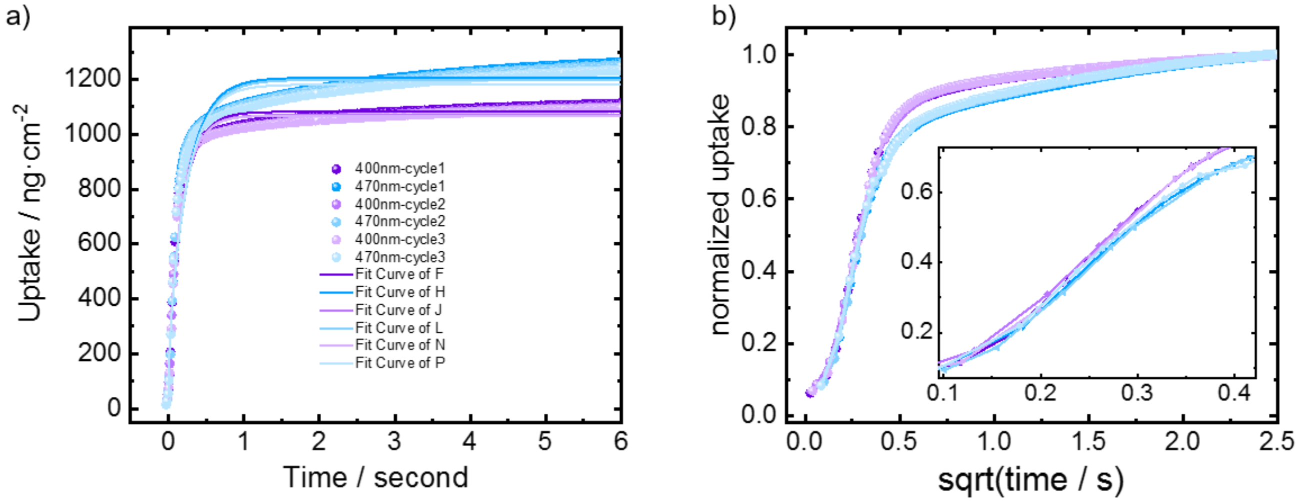

Figure S7. Determining the diffusion coefficients from the transient uptake data. a) The experimental uptake data (spheres) are fitted with the analytical solution for the uptake by an idealized thin film (lines), see equation below. b) The normalized uptakes are plotted *versus* the square-root of time. It can be seen that the uptake by the sample in the stable state (upon 470 nm irradiation, blue data) is somewhat slower than the uptake by the sample in the metastable state (upon 400 nm irradiation, violet data). The color code (same for a and b) is shown in the legend in panel a).

For diffusion-limited uptake processes by a thin homogeneous layer, the normalized uptake can also be calculated by[5]

$$m(t) = 1 - \frac{8}{\pi^2}\sum_{n=1}^{\infty}\frac{\exp\left(-\frac{\pi^2(2n-1)^2 Dt}{4l^2}\right)}{(2n-1)^2},$$

where l is the film thickness l (here 200 nm), t the time t, D the diffusion coefficient and $m(t)$ the transient uptake. This is the analytical solution of Fick's 2nd law. In this equation, any concentration dependence of the diffusion coefficient or any inhomogeneity of the material (like defects or varying film thicknesses) are not considered. The deviation of the experimental data from the ideal uptake behavior are most likely caused by a concentration-dependence of the diffusion coefficient[6] and by potential defects in the MOF structure, like surface barriers, decelerating the uptake process.[7]

References:

[1] W. Danowski, T. van Leeuwen, S. Abdolahzadeh, D. Roke, W. R. Browne, S. Wezenberg, B. L. Feringa, *Nat. Nanotec.* **2019**, *14*, 488-494.

[2] N. Ko, J. Hong, S. Sung, K. E. Cordova, H. J. Park, J. K. Yang, J. Kim, *Dalton Transactions* **2015**, *44*, 2047-2051.

[3] The Cambridge Crystallographic Data Centre entrees are 2215245 for the stable state and 2215244 for the metastable state.

[4] W. Danowski, T. van Leeuwen, S. Abdolahzadeh, D. Roke, W. R. Browne, S. J. Wezenberg, B. L. Feringa, *Nat. Nanotechnol.* **2019**, *14*, 488-494.

[5] a) J. Crank, *The Mathematics of Diffusion*, Clarendon Press, Oxford, **1975**; b) L. Heinke, *Journal of Physics D: Applied Physics* **2017**, *50*, 193004. ; c) J. Kärger, D. M. Ruthven, D. N. Theodorou, *Diffusion in Nanoporous Materials*, Wiley-VCH, **2012**.

[6] L. Heinke, D. Tzoulaki, C. Chmelik, F. Hibbe, J. M. van Baten, H. Lim, J. Li, R. Krishna, J. Kärger, *Phys. Rev. Lett.* **2009**, *102*.

[7] a) C. Li, A. Chandresh, Z. Zhang, S. Moulai, L. Heinke, *Advanced Materials Interfaces* **2022**, *9*, 2101947. ; b) L. Heinke, Z. Gu, C. Wöll, *Nat. Comm.* **2014**, *5*, 4562.
